# Alginate NiFe_2_O_4_ Nanoparticles Cryogel for Electrochemical Glucose Biosensor Development

**DOI:** 10.3390/gels7040272

**Published:** 2021-12-17

**Authors:** Amin Fatoni, Aziz Wijonarko, Mekar Dwi Anggraeni, Dadan Hermawan, Hartiwi Diastuti, Zusfahair

**Affiliations:** 1 Department of Chemistry, Faculty of Mathematics and Natural Sciences, Universitas Jenderal Soedirman, Purwokerto 53122, Indonesia; wijonarkoaziz@gmail.com (A.W.); dadan.hermawan@unsoed.ac.id (D.H.); hartiwi.diastuti@unsoed.ac.id (H.D.); zusfahair@unsoed.ac.id (Z.); 2 Department of Nursing, Faculty of Health Sciences, Universitas Jenderal Soedirman, Purwokerto 53122, Indonesia; mekar.anggraeni@unsoed.ac.id

**Keywords:** alginate, cryogel, electrochemical, glucose biosensor, nickel ferrite nanoparticles

## Abstract

Glucose biosensors based on porous material of alginate cryogel has been developed, and the cryogel provides a large surface area for enzyme immobilization. The alginate cryogel has been supplemented with NiFe_2_O_4_ nanoparticles to improve the electron transfer for electrochemical detection. The fabrication parameters and operational conditions for the biosensor have also been optimized. The results showed that the optimum addition of NiFe_2_O_4_ nanoparticles to the alginate solution was 0.03 g/mL. The optimum operational conditions for the electrochemical detection were a cyclic voltammetry scan rate of 0.11 V/s, buffer pH of 7.0, and buffer concentration of 150 mM. The fabricated alginate NiFe_2_O_4_ nanoparticles cryogel-based glucose biosensor showed a linear response for glucose determination with a regression line of y = 18.18x + 455.28 and R² = 0.98. Furthermore, the calculated detection limit was 0.32 mM and the limit of quantification was 1.06 mM.

## 1. Introduction

Diabetes mellitus is a degenerative disease that causes many deaths. About 43% of the 3.7 million deaths from diabetes mellitus occur before age of 70 years and the percentage of these deaths is higher in developing countries [1]. According to the International Diabetes Federation (IDF), in 2017, the prevalence of diabetes mellitus in the world reached 424.9 million people and is expected to reach 628.6 million in 2045 [2]. Diabetes mellitus is also found as the most comorbidities among individuals died due to COVID-19, especially in Central Java Province, Indonesia, with 39.7% and followed by hypertension at 31.6% (Central Java 2020).

There are preventive measures in place to reduce the number of people with diabetes mellitus. Previous strategies reported for early detection were to determine blood glucose levels with a biosensor [3]. In general, the glucose biosensor uses the enzyme glucose oxidase to catalyze the glucose conversion and the results could be detected electrochemically. The combination of biological sensing elements such as an enzyme and a transducer such as an electrochemical transducer is the main principle of the biosensor for analyte determination [4]. Biosensors have shown several advantages such as being easy to manufacture in small tools (portable), relatively inexpensive, high sensitivity, high selectivity, and making real-time measurements possible. As a result, they have been widely developed and commercialized as analytical tools [5].

The development of biosensors is generally focused on increasing their sensitivity, selectivity, stability, or reducing their production costs. The development strategies could be performed in the biological compound exploration of biological sensing elements such as enzymes, antibodies, cells, supporting materials for biological compound immobilization or the detector improvisation. In developing an enzyme supporting material, a previous study showed that the use of chitosan cryogel increases the surface area of the electrode, thereby increasing the performance of electrochemical biosensors [6]. However, the non-conductive nature of chitosan reduces the electric current when it has been applied with an electrochemical transducer. Various strategies to improve the chitosan conductivity have been explored, such as using carbon nanotubes [7] and grafting with polyaniline [8]. However, it is still challenging to find better enzyme support materials for detecting glucose. Besides chitosan, another biopolymer showed an excellent property as porous supporting material is alginate. Alginate is hydrophilic, biocompatible and biodegradable [9]. Alginate has been used in tissue engineering [10], drug delivery [11], enzyme immobilization [12], and biosensors [13]. Furthermore, the calcium alginate matric for enzyme immobilization could be easily prepared using a simple procedure compared to crosslinking of chitosan. The use of crosslinker agent such as glutaraldehyde is not environmentally friendly, and it could be cytotoxic in case of the biological sensing element using bacteria cell. The green technology using urea-induced gelation for chitosan crosslinking was a relatively complex procedure and resulted in non-stable gel compared to glutaraldehyde crosslinked [14]. Another advantage of alginate matric compared to chitosan is that it has a greater capacity for biomolecule entrapment [15].

Despite the numerous advantages, the use of biomaterial such as chitosan and alginate in the application of biosensors with electrochemical detection has poor mechanical and electrical properties. It has been reported that composites made from biomaterials and nanoparticles overcome these poor electrical properties [7,16]. This research used nickel ferrite nanoparticles to improve the alginate cryogel for glucose biosensor application using electrochemical detection.

## 2. Results and Discussion

### 2.1. NiFe_2_O_4_ Nanoparticles Preparation and Characterization

**Figure 1:**
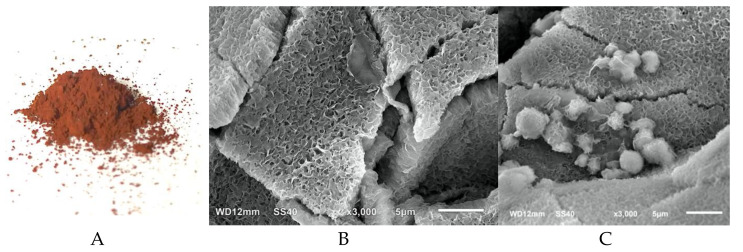
Synthesized NiFe_2_O_4_ nanoparticles showed as a dark brown powder (**A**). The porous structure of cryogel is made of alginate (**B**) and alginate with NiFe_2_O_4_ nanoparticles (**C**).

The NiFe_2_O_4_ nanoparticles were synthesized to improve the performance of the alginate cryogel modified electrode. The NiFe_2_O_4_ nanoparticles were prepared using co-precipitation which was a bottom-up synthesis of nanoparticle from their metal ions to obtain nanosized particles. The co-precipitation method was selected because of its simplicity and ease of obtaining the homogenous size [17,18]. The obtained NiFe_2_O_4_ nanoparticles were a brown powder (Figure 1A). The NiFe_2_O_4_ nanoparticles prepared using the same procedure have been reported to have a particle size of 4.2–5.7 nm observed by transmission electron microscopy and magnetic coercivity of 42–47 Oe [19].

Alginate cryogel was formed by crosslinking sodium alginate under subzero temperature to freeze the water solvent and leave a porous structure [7]. The NiFe_2_O_4_ nanoparticles were entrapped in the alginate structure to facilitate the electron transfer during the redox reaction of the working electrode. The scanning electron microscope image showed that the porous alginate cryogel had a large surface area (Figure 1B,C) with a pore size of about 1–2 micron pores. Subsequently, the alginate NiFe_2_O_4_ nanoparticles cryogel structure and alginate only cryogel showed a similar porous material with an aggregate of NiFe_2_O_4_ nanoparticles on the surface of the alginate NiFe_2_O_4_ nanoparticles cryogel.

### 2.2. Alginate NiFe_2_O_4_ Nanoparticles Modified Electrode Performance

**Figure 2:**
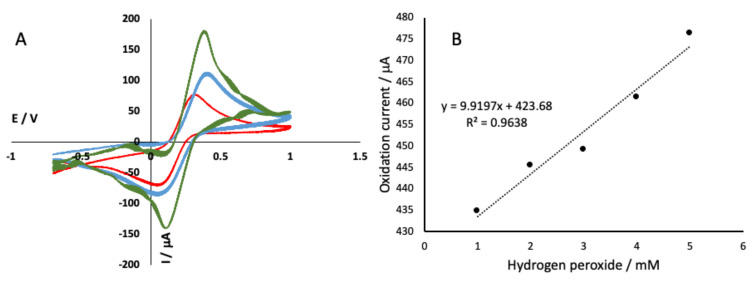
Electron transfer behavior of a bare glassy carbon electrode (**A**, blue line), alginate cryogel modified electrode (**A**, red line), and alginate-NiFe_2_O_4_ nanoparticles cryogel modified electrode (**A**, green line) measured on 10 mM potassium ferricyanide. Alginate-NiFe_2_O_4_ nanoparticles modified electrode showed a linear response for detecting hydrogen peroxide (**B**).

The performance of the alginate-NiFe_2_O_4_ nanoparticles cryogel electrode was tested using potassium hexacyanoferrate and hydrogen peroxide. Potassium hexacyanoferrate was used to observe the electron transfer behavior of each step of electrode modification. The results showed that the glassy carbon electrode with alginate cryogel (Figure 2A, red) had lower oxidation and reduction peaks compared to a bare glassy carbon electrode (Figure 2A, red). The lower oxidation and reduction peaks of alginate cryogel were due to the low conductivity characteristic of alginate [20]. Therefore, strategies to improve the alginate conductivity of the alginate are required. The alginate NiFe_2_O_4_ nanoparticles cryogel showed higher oxidation and reduction peaks (Figure 2A, green), compared to both bar glassy carbon electrode and alginate cryogel modified electrode. The higher oxidation and reduction peaks of the NiFe_2_O_4_ nanoparticles alginate cryogel due to the large surface area of porous cryogel combined with the nickel ferrite nanoparticles. 

It was previously reported that nickel ferrite nanoparticles increase the conductivity of the compound with PANI [21], polypyrrole-chitosan [22] and alginic acid [23]. Besides improving the electron transfer of the alginate cryogel by adding NiFe_2_O_4_ nanoparticles, this nanoparticle with the magnetic properties also makes the alginate cryogel became magnetic alginate cryogel. The magnetic alginate based gels have been reported in many fields such as biosensors [24], drug delivery [25] and tissue engineering [26]. The magnetic materials embedded in the gels provide unique features such as responding to the applied magnetic fields, inducing shape changes and modifying the mechanical properties [27]. The nanoparticle of NiFe_2_O_4_ provides magnetism of small size ferromagnetic (superparamagnetism) showed unique properties that could enhance the biosensor sensitivity and allow rapid detection of various analytes [28]. Therefore, the increase of the redox peaks observed in this research can also be caused by the super magnetism of nanoparticles added to the alginate gels.

Hydrogen peroxide was first used to simulate the glucose biosensor, since the use of glucose oxidase enzyme would result in hydrogen peroxide which was eventually detected by electrochemical detection. The electrochemical method used was cyclic voltammetry, which is a cyclic method that describes the movement of electrons due to a reduction and oxidation reaction occurring on the surface of the working electrode. The results showed a linear response of hydrogen peroxide determination (1–5 mM, in phosphate buffer) using alginate-NiFe_2_O_4_ nanoparticles modified electrode performed using cyclic voltammetry (Figure 2B).

### 2.3. NiFe_2_O_4_ Nanoparticles Addition Optimization

NiFe_2_O_4_ nanoparticles were added to the alginate cryogel to improve the electron transfer properties. However, the addition of nanomaterials could be increasing the cost in further application. Therefore, finding the best condition with the lowest number of nanoparticles addition was important but showed the optimal electron transfer properties. 

**Figure 3:**
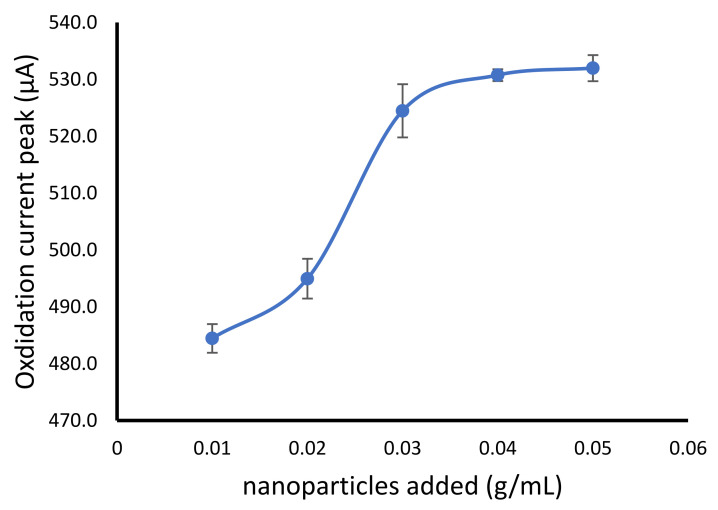
Effect of NiFe_2_O_4_ nanoparticles addition to alginate cryogel on the increase of oxidation peak of hydrogen peroxide.

The NiFe_2_O_4_ nanoparticles was added in various final concentration of 0.01, 0.02, 0.03, 0.04 and 0.05 g/mL of sodium alginate solution. The result showed the increase of oxidation current with the addition of NiFe_2_O_4_ nanoparticles from 0.01 to 0.03 g/mL. However, the higher amount of the nanoparticles did not show a significant oxidation current change (Figure 3). The nanoparticle composite in alginate polymers was generally prepared in the composition of 0–10% (*w*/*v*) such as in the alginate-magnetic nanoparticles [29], alginate silver nanoparticles [30], alginate-Fe_3_O_4_ nanoparticles [31] and alginate multi-walled carbon nanotubes [32].

### 2.4. Effect of Cyclic Voltammetry Scan Rate

**Figure 4:**
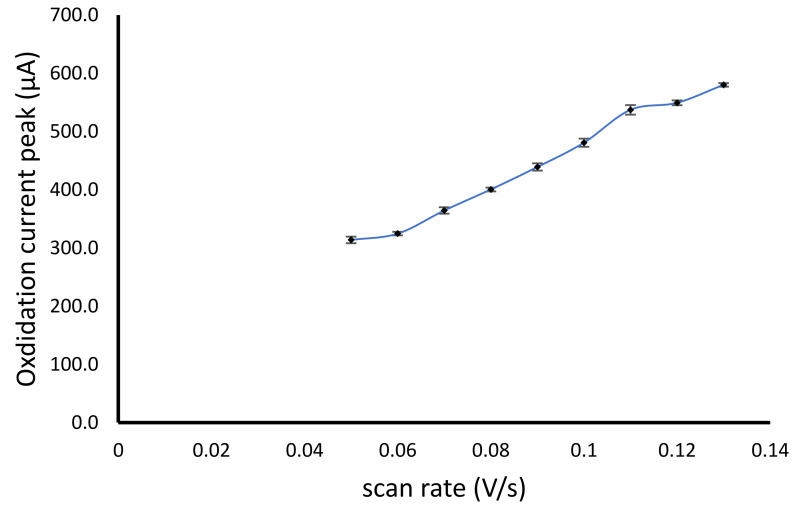
Scan rate optimization of the cyclic voltammetry method for hydrogen peroxide determination using modified alginate NiFe_2_O_4_ nanoparticles cryogel electrode.

The CV method for the determination of hydrogen peroxide using modified alginate NiFe_2_O_4_ nanoparticles has been studied the effect of scan rate using phosphate buffer pH 7.0 with a concentration of 100 mM. The scan rate range used was 0.05–0.13 V/s. The increase in the scan rate causes the increase of electron transfer per second, leading to the accumulation of electrolyte ions. Therefore, each increase in the scan rate would increase the oxidation peak current, making it difficult to obtain the optimum condition of the scan rate, similar to the previous report [33]. However, an increase in scan rate with the increase in the oxidation peak current from 0.06 to 0.11 V/s showed a higher current increase, while the higher scan rate of more than 0.11 V/s showed a lower increase in the oxidation peak current (Figure 4). 

### 2.5. Effect of Buffer pH and Concentration on the Detection of Hydrogen Peroxide

**Figure 5:**
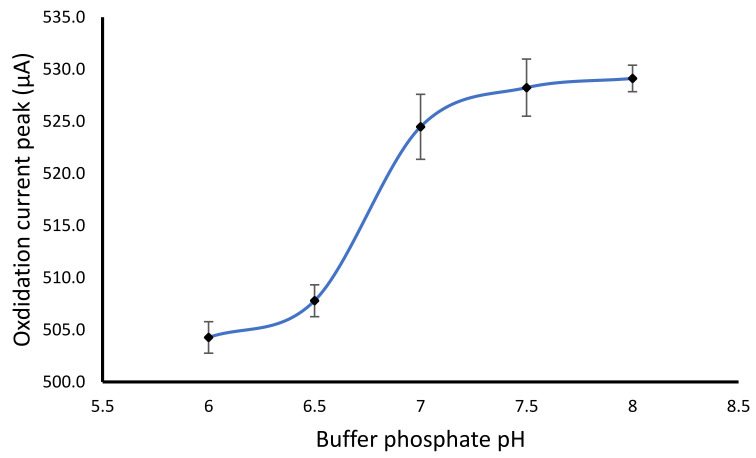
Effect of buffer pH on the hydrogen peroxide oxidation peak using the modified alginate-NiFe_2_O_4_ nanoparticles cryogel electrode.

The buffer pH was optimized using 0.1 M phosphate buffer with various pH of 6.0, 6.5, 7.0, 7.5 and 8.0. The result showed that the optimum pH for the determination of hydrogen peroxide was at a pH of 7.0 (Figure 5). The increase of buffer pH from 6.0 to 7.0 showed an increase of oxidation current peak. However, the higher pH of more than 7.0 did not significantly increase in the current oxidation peak. The pH of 7.0 was also selected based on the glucose oxidase enzyme having an optimum pH of 7.0 when applied as glucose biosensor [7]. In some cases, the change of pH can lead to a change in oxidation or reduction peak [34], which may be favorable to improve the selectivity. In this study, various pH showed similar oxidation peak potential but different their peak height.

**Figure 6:**
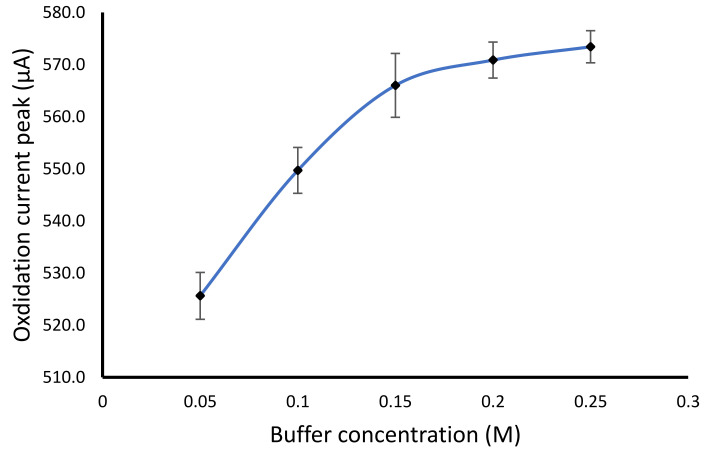
Buffer concentration effect on the hydrogen peroxide determination using the modified electrode.

The concentration of the phosphate buffer influences the redox behavior of hydrogen peroxide, since the electron transfer highly depends on the electrolyte concentration in the medium. The result showed an increase in the buffer concentration from 50 to 150 mM, while the higher buffer concentration did not show a significant increase (Figure 6). The buffer concentration of the buffer was an important factor affecting the sensitivity of the biosensor. The concentration of the buffer causes a change in the capacity of the ionic form of the substance in the solution. The higher the buffer concentration, the more free ions from the buffer salt contained in the solution, thus increasing the current value. Optimal buffer concentration could provide a high sensitivity of the biosensor.

### 2.6. Glucose Determination Using Fabricated Biosensor

The glucose determination was performed with the glucose oxidase enzyme entrapped in the alginate NiFe_2_O_4_ nanoparticles cryogel. The electrochemical measurement was based on the determination hydrogen peroxide which results from the enzymatic reaction of glucose catalyzed by glucose oxidase in the following redox reaction:

(1) $$Glucose+O_{2}\overset{glucoseoxidase}{\to }H_{2}O_{2}+gluconicacid$$

(2) $$H_{2}O_{2}\overset{modifiedelectrode}{\to }O_{2}+2H^{+}+2e^{-}\left(ox\right)$$

(3) $$H_{2}O_{2}+2H^{+}+2e^{-}\overset{modifiedelectrode}{\to }2H_{2}O\left(red\right)$$

**Figure 7:**
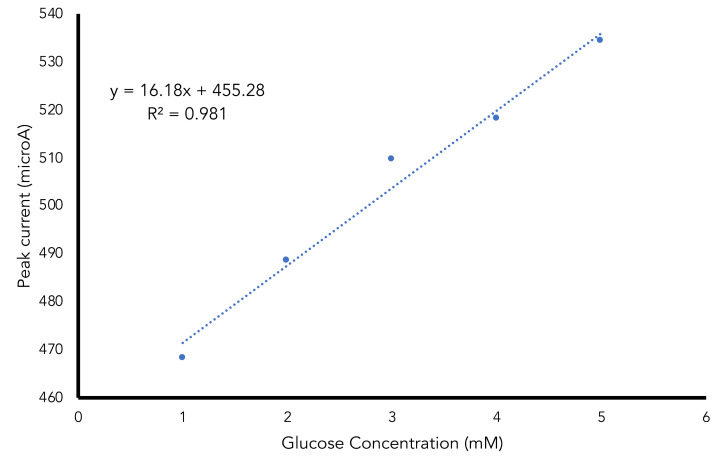
Glucose determination using fabricated alginate NiFe_2_O_4_ nanoparticles cryogel biosensor.

The result showed that the peaks current was increased linearly with the glucose concentration with the regression equation of y = 16.18x + 455.28 and R^2^ of 0.981 (Figure 7). The calculated limit of detection and limit of quantification were 0.32 mM and 1.06 mM respectively. The fabricated electrochemical alginate-NiFe_2_O_4_ nanoparticles cryogel based glucose biosensor was more sensitive compared to colorimetric alginate-based glucose biosensor [13] and near-infrared alginate-based glucose biosensor [35].

## 3. Conclusions

Alginate NiFe_2_O_4_ nanoparticles composite as supporting material in the fabricated glucose biosensor showed a high surface area and good electron transfer using an electrochemical detector. The optimal conditions obtained were the addition of NiFe_2_O_4_ nanoparticles of 0.03 g/mL sodium alginate solution, a scan rate of 0.11 V/s, phosphate buffer pH of 7.0 and buffer concentration of 150 mM. The determination of the glucose using immobilized GOD enzyme showed a linear response with regression equation of y = 16.18x + 455.28 and R ² of 0.98. The limit of detection obtained was 0.32 and the limit of quantification was 1.06 mM. 

## 4. Materials and Methods

### 4.1. Materials

Alginic acid sodium salt from brown algae (BioReagent, Sigma-Aldrich, St. Louis, MI, USA), glucose oxidase from *Aspergillus niger* (type II, ≥15,000 U/g solid, Sigma-Aldrich, St. Louis, MI, USA), nickel(II) chloride (NiCl_2_·6H_2_O) Merck KGaA, Darmstadt, Germany), iron(III) chloride (FeCl_3_·6H_2_O) (Merck KGaA, Darmstadt, Germany), glucose anhydrous (≥98.0%) (Sigma), acetic acid (CH_3_COOH) (Merck KGaA, Darmstadt, Germany), hydrogen peroxide (H_2_O_2_) 30% (Merck KGaA, Darmstadt, Germany), sodium hydroxide (NaOH) (Merck KGaA, Darmstadt, Germany), disodium hydrogen phosphate (Merck KGaA, Darmstadt, Germany) and sodium dihydrogen phosphate (Merck KGaA, Darmstadt, Germany).

### 4.2. Apparatus and Measurements

Scanning electron microscopy (SEM) (JSM-6510 LA, JEOL, Tokyo, Japan), operating at 15 kV, was used to examine the morphology of the alginate nanoparticle cryogel. The electrochemical analysis was performed using a three-electrode system with a glassy carbon electrode as a working electrode and Ag/AgCl as a reference electrode and platinum wire as a counter electrode. The electrochemical measurements were carried out using Rodeostat Potentiostat (IORodeo Smart Lab Technology, Pasadena, CA, USA). 

### 4.3. NiFe_2_O_4_ Nanoparticles Preparation

Nickel ferrite nanoparticles have been synthesized using the co-precipitation method [36] with NiCl_2_·6H_2_O and FeCl_3_·6H_2_O as ion providers Ni^2+^ and Fe^3+^. The mole fraction ratio used was 1:2, by dissolving 1.188 g of NiCl_2_·6H_2_O in 20 mL distilled water and 2701 g of FeCl_3_·6H_2_O in 20 mL of distilled water in a separate glass beaker. The two solutions were then mixed homogeneously. The mixture of Ni and Fe was then dropwise slowly in the precipitation agent of NaOH under stirring (1000 rpm) at 85 °C for 60 min. The variation of NaOH concentrations used were 3, 5, 7, 9 and 11 M. The resulting nanoparticles were then precipitated continued by washing distilled water for approximately seven times of 50 mL. The precipitated nanoparticles obtained were dried at 90 °C. The brown nanoparticles powder was then used for further procedures.

### 4.4. Alginate Cryogel Electrode Preparation

Sodium alginate solution was prepared by dissolving 2.0 g of alginate in phosphate buffer (100 mM, pH 7) to get 100 mL of solution. The 50 μL of alginate solution was then drop on the glassy carbon working electrode (3 mm diameter). The electrode with alginate cover was immerse in 2 M CaCl_2_ solution and allowed for 30 min to make the crosslink layer of Ca-alginate on the electrode. The electrode was then kept in the freezer at −20 °C for 12 h to continue the crosslinking reaction with the freezing condition for cryogel forming. 

Alginate NiFe_2_O_4_ nanoparticles were prepared using similar procedure above with the addition of NiFe_2_O_4_ to the alginate solution with the concentration of 0.1, 0.2, 0.3, 0.4 and 0.5 g per 100 mL alginate solution. The mixture was then dropped in on the glassy carbon electrode and further the procedure of alginate cryogel modified electrode. The modified electrode with alginate cryogel and alginate NiFe_2_O_4_ has been tested using K_3_[Fe(CN)_6_] and H_2_O_2_ (in 100mM phosphate buffer, pH of 7.0) solution by cyclic voltammetry method.

### 4.5. Cyclic Voltammetry Optimization

The optimal condition of the electrochemical cell using cyclic voltammetry (−1.0 to 1.0 V, 3 scans) were performed by variating the scan rate of 0.05, 0.06, 0.07, 0.08, 0.09, 0.10, 0.11, 0.12 and 0.13 mV/s. The solution used was H_2_O_2_ 5 mM in phosphate buffer pH 7.0. The best scan rate with the minimum background current was then selected for further study. 

### 4.6. Buffer pH and Concentration Optimization

The buffer pH and concentration were optimized in the H_2_O_2_ determination with a variation of pH 6.0 to 8.0 with the various concentrations of 50, 100, 150, 200 and 250 mM. The cyclic voltammetry conditions included the optimized parameters obtained.

### 4.7. Glucose Determination Using Modified Electrode

Glucose oxidase as biological sensing element was prepared in buffer solution (pH 7.0, concentration 50 mM). The enzyme was immobilized in the working electrode by entrapping method. The glucose oxidase solution was then added to the alginate solution (20 μL/1000 μL) with the total enzyme activity of 50 U per modified electrode made of 50 μL solution of alginate-NiFe_2_O_4_ nanoparticles. The mixture was then dropped on the working electrode surface, dipped in the crosslinking agent and kept in the freezer for cryogelation. The modified electrode was then tested to detect glucose standard solution under the optimal condition. Linearity, the limit of detection and limit of quantification were then calculated from the series concentration of glucose determined.

## Data Availability

The data presented in this study are available on request from the corresponding author.
